# The effects of standardised versus individualised seat height on 1-minute sit-to-stand test performance in healthy individuals: a randomised crossover trial

**DOI:** 10.1007/s00421-023-05174-8

**Published:** 2023-03-17

**Authors:** Manuel Kuhn, Sibylle Vollenweider, Christian F. Clarenbach, Dario Kohlbrenner

**Affiliations:** 1 Faculty of Medicine, University of Zurich, Zurich, Switzerland; 2 Department of Pulmonology, University Hospital Zurich, Zurich, Switzerland

**Keywords:** 1-minute sit-to-stand test, Seat height, Functional exercise testing, Exercise capacity, Morphology

## Abstract

**Purpose:**

We aimed to (i) investigate differences in 1-minute sit-to-stand test (1MSTST) performance (i.e., the number of repetitions) between a standardised modality (i.e., starting from a conventional chair with 46 cm seat height) and an individualised modality (i.e., starting with a knee joint flexion angle of 90°), and to (ii) quantify the influence of tibia and femur length on 1MSTST performance.

**Methods:**

Healthy participants were recruited for this randomised crossover study, performing each 1MSTST modality twice in a randomised order. The primary outcome was the number of repetitions in the 1MSTST. Secondary endpoints were the acute responses in peripheral oxygen saturation, heart rate, and leg fatigue and dyspnoea. Additionally, we investigated correlations of performance with knee extensor strength in both modalities.

**Results:**

Thirty participants were recruited and completed the study. They achieved significantly less repetitions in the standardised 1MSTST compared to the individualised 1MSTST (B = − 12.1, 95% confidence interval [95% CI] =  − 14.8/− 9.4, p < 0.001). We found a significant effect of femur length on 1MSTST performance (B = − 1.6, 95% CI = − 2.6/− 0.7, p = 0.01), tibia length showed significant interaction with the 1MSTST modality (B = 1.2, 95% CI = 0.2/2.2, p = 0.03).

**Conclusion:**

An individualisation of the 1MSTST starting position to 90° knee flexion angle leads to more repetitions compared to the traditional starting position. The higher repetition count is explained by controlling for differences in tibia length. We recommend individualisation of the 1MSTST, enabling more valid comparisons across populations and study samples.

**Trial registration number:**

http://www.ClinicalTrials.gov, NCT04772417.

**Trial registration date:**

February 26, 2021.

## Introduction

Quantifying endurance exercise capacity, cardiopulmonary exercise testing is defined as the gold standard (Franklin and American College of Sports 2000). However, activities of daily living and sports performance cannot be limited to only endurance exercise capacity. Therefore, more functional, inexpensive, rapidly available and easy tests are applied in the broad sports community and in clinical practice. One of the most frequently used field tests is the 1-minute sit-to-stand test (1MSTST), quantifying functional exercise capacity by the number of sit-to-stand (STS) manoeuvres achieved during 1 min (Bohannon and Crouch 2019). Using STS manoeuvres as a marker for functional exercise capacity is not a novel approach. Csuka and McCarthy first described these applications in 1985 (Csuka and McCarty 1985). Nowadays, numerous versions of STS tests have been described, ranging from a few seconds (5 repetitions) to a few minutes. However, evidence suggests the 1-min version as the best option (Bohannon and Crouch 2019). The 1MSTST was validated in various clinical populations and reference values are available (Vaidya et al. 2017; Strassmann et al. 2013; Radtke et al. 2017).

It is appealing to perform functional exercise capacity testing with little and easily accessible material, such as a chair and a stopwatch. However, this simplicity may be a source for bias and inaccuracy regarding performance quantification. More precisely, seat height of conventional chairs for 1MSTSTs ranges between 44.5 and 48 cm. Depending on body height of participants, this results in significant inter-individual knee joint angle variability during testing (Chorin et al. 2015). There is evidence from biomechanical studies that variability in knee joint angle changes ground reaction forces during STS manoeuvres significantly (Chorin et al. 2015). Furthermore, requirements on knee extensor torque increase significantly when lowering seat height below 115% of knee height (Rodosky et al. 1989; Hughes et al. 1996). This ultimately leads to non-generalisability of 1MSTST performance across populations and study samples.

Biomechanical evidence suggests standardisation of the knee joint flexion angle to 90° in the starting position for STS evaluations in order to generate more reliable data (Chorin et al. 2015; Schenkman et al. 1996). Angles below 90° make the movement more demanding and may ultimately result in an unsuccessful attempt in performance-impaired individuals (Schenkman et al. 1990; Munro et al. 1998). However, this suggestion is to date not applied in clinical practice and only a single study, investigating patients with chronic obstructive pulmonary disease, exists comparing the two seat heights (Zumbrunnen et al. 2022).

Since knee joint angles in seated position are mainly determined by tibia length, we hypothesized that controlling for this factor would prevent over- or underestimation of functional exercise capacity in 1MSTST data. Allometric scaling may do controlling (Jaric et al. 2005), however, applying equal test environments for all participants may be the best approach.

Thus, we aimed to (i) investigate differences in 1MSTST performance (i.e., the number of repetitions) between a standardised modality (i.e., starting from a conventional chair) and an individualised modality (i.e., starting with a knee joint flexion angle of 90°), and to (ii) quantify the influence of tibia and femur length on 1MSTST performance.

## Material and methods

Healthy participants aged 18 years and older were deemed eligible for this study. We excluded all participants who had a pre-diagnosed clinical condition, experienced a respiratory tract infection within the last 2 weeks, were either current or previous smokers who quit less than 1 year ago, and were unable to perform or had pain during the STS manoeuvres. All participants were advised to refrain from vigorous physical activities for at least 24 h before the study procedure. In addition, they were requested to not ingest fatty meals and caffeine for at least four hours before the commencement of the study procedure.

We performed a randomised crossover study at the Department of Pulmonology, University Hospital Zurich, Zurich, Switzerland. Participants attended a single study visit and were randomly assigned to the experimental sequence (i.e., either performing a 1MSTST from standard seat height first or performing a 1MSTST from individualised seat height first) by computerised randomisation.

We conducted this study in accordance with the Declaration of Helsinki (2023), the principles of Good Clinical Practice, the Human Research Act and the Human Research Ordinance (Human Research Act (HRA) 2023). All subjects provided written informed consent. The Ethics Committee of the Canton of Zurich approved the study (EK-ZH-NR: 2020-02945), and the study is registered on www.ClinicalTrials.gov (NCT04772417).

The study is reported in accordance with the Consolidated Standards of Reporting Trials (CONSORT) 2010 statement: extension to randomised crossover trials (Dwan et al. 2019).

The standardised 1MSTST was conducted following published protocols (Radtke et al. 2016; Crook et al. 2017). A conventional chair without armrests and a seat height of 46 cm was used. For safety reasons, the chair was placed against a wall. Participants were instructed to stand up and sit down as many times as possible at a self-selected speed for 1 min. The assessor counted the number of repetitions. No verbal encouragement was given during the test, but after 45 s participants were told that there were fifteen seconds left. Participants were allowed to take a break at any time during the test if needed. However, they were instructed to resume movement as soon as possible. For a valid repetition, the knees had to be fully extended when standing up and when sitting down, the buttocks had to be in contact with the chair. The subjects were asked to rest their arms on their hips, avoiding them to support the movement.

The individualised 1MSTST was performed according to the same protocol and instruction guide as the standardised 1MSTST. However, we adjusted seat height to ensure a knee flexion angle of 90° was present in the starting position. We aimed to elicit maximal performance in each participant and therefore did not restrict ankle dorsiflexion. Instead, we applied the following procedure: (i) adjusting seat height, (ii) participant performed three trial repetitions aiming to position their feet at the subjectively best position, (iii) readjustment of seat height if required.

**Fig. 1:**
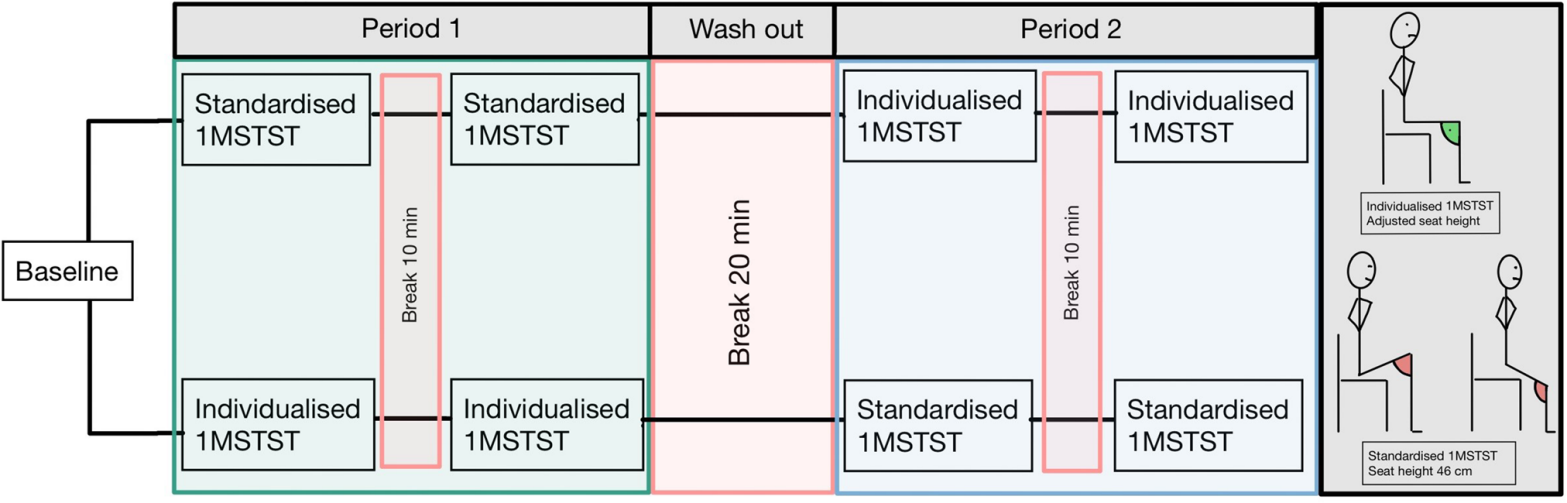
Experimental day. Participants attended a single experimental day and were randomly assigned to the sequence of tests (i.e., the order of Period 1 and Period 2). The standardised 1MSTST was done on a standard chair with seat height 46 cm; the individualised 1MSTST was done from a seat height adjusted to a knee flexion angle of 90° in the starting position. *1MSTST* 1-minute sit-to-stand test

During the experimental day, both modalities of the 1MSTST were measured twice, controlling for a possible learning effect (Radtke et al. 2016). Adequate rest was provided between the tests, allowing heart rate and peripheral oxygen saturation (SpO_2_) a return to baseline values. Within the two tries of the same modality, there was a break of at least 10 min. Between the two modalities, there was a break of 20 min. The experimental day is graphically displayed in Fig. 1.

The primary outcome of this investigation was the number of repetitions in the 1MSTST. As secondary endpoints, we collected data on the acute physiological response to the 1MSTSTs; SpO_2_ and heart rate were measured with a portable pulse oximeter (Nellcor PM10-N, Medtronic, Minneapolis, USA). In addition, we assessed leg fatigue and dyspnoea using the modified Borg scale ranging from 0 (indicating no leg fatigue or dyspnoea at all) to 10 (indicating maximal leg fatigue or dyspnoea) (Borg et al. 2010). Last, we measured knee extension torque on both legs using handheld dynamometry (MicroFET2, Hoggan Industries, West Jordan, UT, USA). The dynamometry was performed in accordance with a standard operating procedure, collecting three reproducible measurements with the “break” technique (Douma et al. 2014). Participants sat on a treatment bench with their feet off the floor. They placed their hands on the bench’s edges and their hips were fixed to the bench with a strap to ensure standardisation and maximal effort. A single rater collected all torque data and we minimised risk of bias further by using a fixation attached to the dynamometer. We used the highest values from both sides for analysis. The results were expressed as torque in newton metres (Nm).

Regarding demographical and morphological variables, we measured body weight, body height, femur length, and tibia length. Two blinded assessors quantified femur and tibia length and the mean value of these measurements was used for analysis. Tibia and femur length were measured by tape measure in a supine, relaxed position. To evaluate tibia length, the lateral knee joint gap and the lower edge of the malleolus lateralis were used as landmarks. Whereas femur length was measured using the trochanter major and the lateral knee joint gap as landmarks.

To ensure all participants were able to perform at their maximal capacity, we asked them to rate their current health status on the Feeling Thermometer from 0 (worst possible status) to 100 (perfect health) before beginning with the experiment (Munro et al. 1998).

All results are shown as mean (SD) unless stated otherwise, the distribution of the variables was determined visually using quantile–quantile plots. A two-sided p value < 0.05 was considered statistically significant.

We analysed the effect of the 1MSTST modality on the number of repetitions (i.e., the primary outcome) using linear mixed regression modelling with random intercepts. We adjusted for carry-over and period effects. Regarding the dependent variable, we proceeded with the try in each modality that elicited the highest number of repetitions. To estimate the influence of morphology (i.e., length of femur and tibia), we applied the stepwise backward elimination procedure to linear mixed regression modelling with interaction terms and random intercept.

Regarding the analysis of the secondary outcomes, we used descriptive statistics. The acute responses to each modality were described by calculating the differences in SpO_2_, heart rate, and Borg ratings between pre- and post-test values. Knee extension torque was correlated with the number of repetitions in each 1MSTST modality using Pearson’s product-moment correlation. The strength of correlation was classified in accordance with published recommendations as negligible (0 ≤ r ≤ 0.29), low (0.3 ≤ r ≤ 0.49), moderate (0.5 ≤ r ≤ 0.69), high (0.7 ≤ r ≤ 0.89) and very high (0.9 ≤ r ≤ 1) correlation (Evans 1996). We used the highest out of the three reproducible dynamometry attempts per side and applied allometric scaling to correct for the effect of body weight on performance in both dynamometry and the 1MSTST (Jaric et al. 2005). For dynamometry, we divided torque by body weight, and for the 1MSTST, we divided the number of repetitions by body weight^−1/3^ (Jaric et al. 2002, 2005).

Our sample size determination was based on the evidence that regression modelling requires at least 10 participants per independent variable included in the final model (Harrell 2015). Our model aimed to include femur length, tibia length, and 1MSTST modality as independent variables. As such, we aimed to include 30 participants.

All analyses were performed with R version 4.0.3 for Windows (R Core Team 2022, R Foundation for Statistical Computing, Vienna, Australia).

## Results

**Fig. 2:**
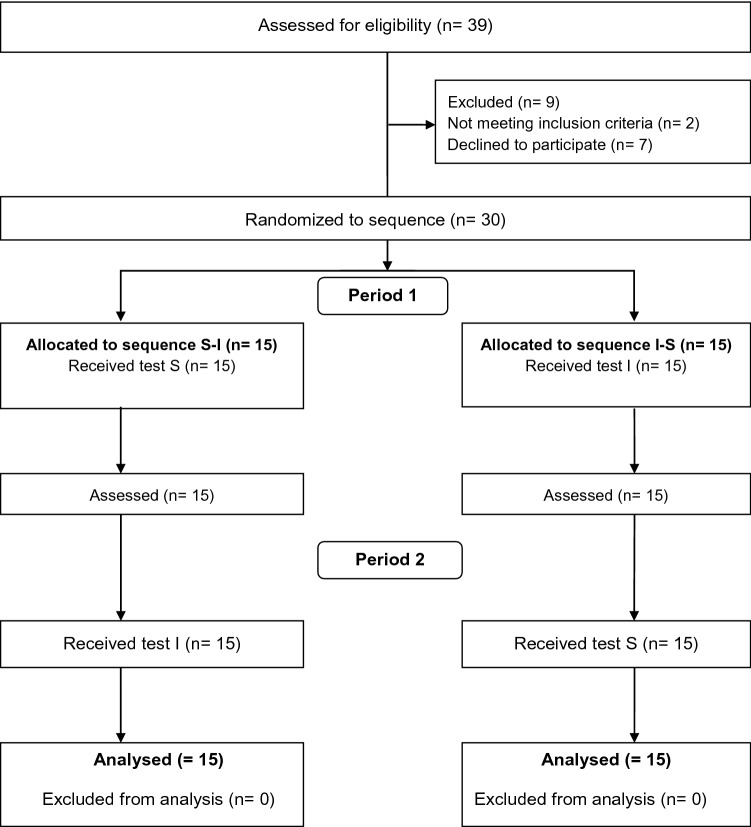
Study participant flow diagram. Period describes which order of tests the participants were randomised to; *S-I* standardised (i.e., 46 cm seat height)–individualised (i.e., 90° knee flexion angle in starting position); *I-S* individualised–standardised; *S* standardised; *I* individualised

Thirty participants (15 men and 15 women) were included and completed the study. For the detailed participant flow, consult Fig. 2. The sample was 32 (9) years of age, had a body-mass index of 23.6 (2.9), a femur length of 44.5 (3.1) cm and a tibia length of 43.7 (2.4) cm. For further participant characteristics, see Table 1.

**Table 1:** Participant characteristics

Variable	Value
n	30
Age, years	32 (9)
Sex, female, n (%)	15 (50)
Weight, kg	72.1 (11.9)
Height, cm	174.4 (7.1)
BMI	23.6 (2.9)
Length femur, cm	44.5 (3.1)
Length tibia, cm	43.7 (2.4)
Knee extension torque right, Nm	517.2 (147.4)
Knee extension torque left, Nm	471.5 (128.9)
Repetitions standardised 1MSTST	57 (10)
Repetitions individualised 1MSTST	70 (11)

Data are mean (SD) or n (%)

*BMI* body-mass-index; *1MSTST* 1-minute sit-to-stand test; *Nm* Newton metres

**Fig. 3:**
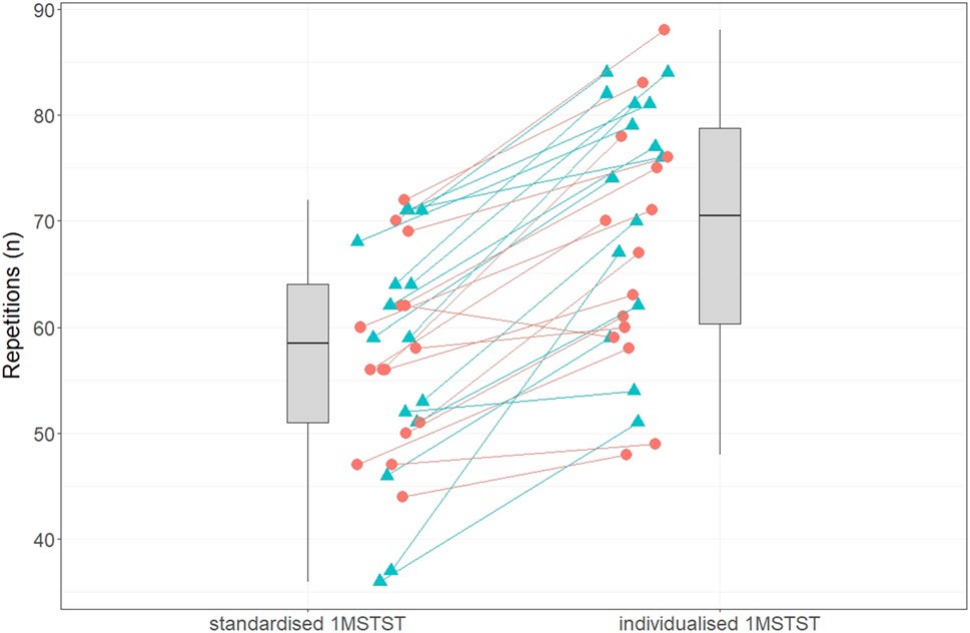
Repetitions performed in the standardised and individualised 1MSTST. Individual data is stratified for females (red circles) and males (blue triangles). *1MSTST* 1-minute sit-to-stand test

In the standardised 1MSTST, participants achieved 57 (10) repetitions. In contrast, they achieved 70 (11) repetitions in the individualised 1MSTST. Individual repetition counts for both modalities are displayed in Fig. 3; all but one participant achieved more repetitions in the individualised 1MSTST. Linear mixed regression modelling with random intercept showed that repetitions in the standardised 1MSTST were significantly less compared to the individualised 1MSTST (B = − 12.1, 95% CI = − 14.8/− 9.4, p < 0.001).

Linear mixed regression modelling with stepwise backward elimination showed a significant effect of femur length on 1MSTST performance (B = − 1.6, 95% CI = − 2.6/− 0.7, p = 0.01), tibia length showed significant interaction with the 1MSTST modality (B = 1.2, 95% CI = 0.2/2.2, p = 0.03).

**Fig. 4:**
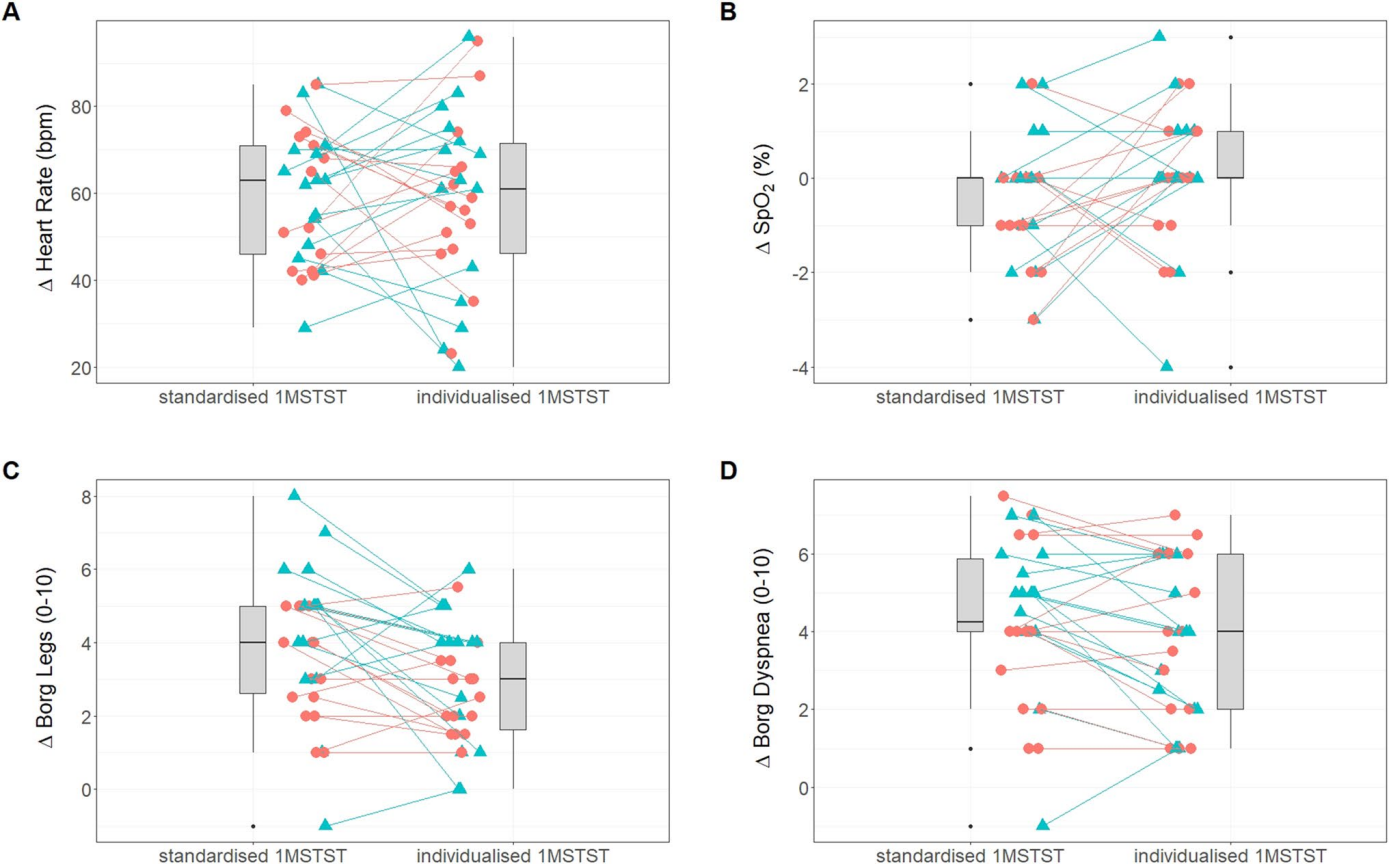
Acute physiological responses in the standardised and the individualised 1MSTST. Displayed as individual changes (Δ). Individual data is stratified for females (red circles) and males (blue triangles). The panels show responses in **a** heart rate; **b** SpO_2_; **c** perception of leg fatigue; **d** perception of dyspnoea. *1MSTST* 1-minute sit-to-stand test; bpm, beats per minute; *SpO*_*2*_ peripheral oxygen saturation

In the standardised 1MSTST SpO_2_ decreased by 0.4 (1.4) %, heart rate increased by 54 (15) beats per minute (bpm), leg fatigue increased by 4 (2) points, and dyspnoea increased by 4 (2) points. In the individualised 1MSTST SpO_2_ decreased by 0.1 (1.4) %, heart rate increased by 56 (18) bpm, leg fatigue increased by 3 (2) points, and dyspnoea increased by 4 (2) points. Table 2 provides the pre-, post-, and delta-values for both test modalities and Fig. 4 displays individual participant changes in all parameters.

**Table 2:** Acute physiological response to the standardised and individualised 1MSTST

Standardised 1MSTST			
	Pre-test	Post-test	Δ
SpO_2_ (%)	98 (1.1)	97 (1.1)	0.4 (1.4)
Heart rate (bpm)	85 (15)	139 (17)	54 (15)
Borg dyspnoe (0–10)	0 (1)	5 (2)	4 (2)
Borg leg (0–10)	1 (1)	5 (2)	4 (2)

Individualised 1MSTST			
	Pre-test	Post-test	Δ
SpO_2_ (%)	98 (0.8)	98 (1.3)	0 (1.4)
Heart rate (bpm)	85 (14)	141 (20)	56 (18)
Borg dyspnoe (0–10)	0 (1)	4 (2)	4 (2)
Borg leg (0–10)	1 (1)	4 (2)	3 (2)

Data are mean (SD)

*1MSTST* 1-minute sit-to-stand test; *SpO*_*2*_ peripheral oxygen saturation; *bpm* beats per minute; *pre-test* resting value immediately before test; *post-test* value immediately after test; Δ delta

Knee extension torque was 517.2 (147.4) Nm for the right leg and 471.5 (128.9) Nm for the left leg.

**Fig. 5:**
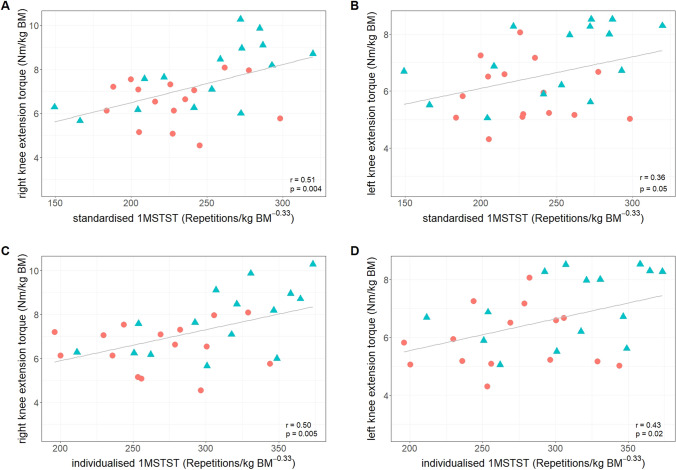
Correlations between allometrically scaled 1MSTST repetitions and knee extension torque. Individual data is stratified for females (red circles) and males (blue triangles). **a** standardised 1MSTST and right knee extension torque; **b** standardised 1MSTST and left knee extension torque; **c** individualised 1MSTST and right knee extension torque; **d** individualised 1MSTST and left knee extension torque. *1MSTST* 1-minute sit-to-stand test; *BM* body mass; *Nm* Newton metres

In the standardised 1MSTST, knee extension torque of the right leg showed moderate (r = 0.51, 95% CI = 0.18/0.73, p = 0.004), and the left leg low (r = 0.36, 95% CI = -− 0.0/0.64, p = 0.05) correlation with the number of repetitions in the 1MSTST. In the individualised 1MSTST, knee extension torque of the right leg showed moderate (r = 0.50, 95% CI = 0.17/0.73, p = 0.005), and the left leg low (r = 0.43, 95% CI = 0.08/0.68, p = 0.02) correlation with the number of repetitions in the 1MSTST. The correlations are visualised in Fig. 5.

## Discussion

We report on the first study comparing 1MSTST performance from a standardised starting position (i.e., 46 cm seat height) versus an individualised starting position (i.e., starting with a knee flexion angle of 90°) in healthy participants. We found that the individualised 1MSTST led to a significant increase in repetitions compared to the standardised 1MSTST. As hypothesised, 1MSTST performance was explained by morphology (i.e., tibia and femur length). Our investigation showed that the individualised 1MSTST controls for the effect of tibia length on performance.

In our study, participants achieved an average of 12 more repetitions in the individualised 1MSTST than in the standardised 1MSTST. There was significant influence of both femur and tibia length on performance. However, the individualised 1MSTST is only able to control for length differences in the tibia. Thus, the difference between the modalities is explained by controlling for tibia length differences. Accordingly, our findings confirm previous hypotheses based on biomechanical investigations (Takai et al. 2009; Yamada and Demura 2004; Chorin et al. 2015a). It is of vast clinical importance to show that the biomechanical observations, obtained from only few STS manoeuvres, remain valid when performing manoeuvres at maximal cadence during a prolonged time. Nevertheless, it remains to be investigated how controlling for femur length and torso length can be done effectively. Meanwhile, we recommend the use of our approach with a knee flexion angle of 90° in starting position, achieved through adjustments of seat height while the participant chooses foot position deliberately according to comfort, as a new standard operating procedure. The individualised 1MSTST is feasible, easy to use, and can be standardised. Of note, individualising the test led to a heightened seating height in all of our participants. Therefore, future studies should make sure to include also shorter participants, describing the effects on performance when seat height is lowered. Obviously, we may hypothesise that in these cases repetition count would drop.

Acute physiological responses (i.e., heart rate and SpO_2_) were largely similar between the standardised and the individualised 1MSTST. From this finding, we conclude that participants were able to elicit maximal effort in both 1MSTST modalities. In the standardised 1MSTST, a trend towards desaturation was visible. However, the difference between the modalities might not be clinically relevant (Palange et al. 2007; Rodrigues et al. 2013). Essentially, in samples of healthy participants no relevant desaturation is expected, and we encourage further research investigating the acute responses to the individualised 1MSTST in clinical populations prone to exercise induced desaturation (e.g., chronic heart or lung disease populations). First data in chronic obstructive pulmonary disease is available, showing no clinically relevant differences between the test modalities (Zumbrunnen et al. 2022).

Our conclusion on the ability of participants to elicit maximal performance in both 1MSTST modalities was supported by the subjective ratings on the modified Borg scale. While sensations of dyspnoea were largely similar between the standardised and the individualised 1MSTST, there was a tendency towards lower sensations of leg fatigue in the individualised 1MSTST. This difference might be clinically relevant and would thus require confirmation in further studies.

It is well known that 1MSTST performance is associated with knee extension strength (Bohannon and Crouch 2019; Kohlbrenner et al. 2020; Bhardwaj et al. 2021). Performing a standardised 1MSTST as a tall person might exacerbate this association and hamper comparison of performance with individuals of a shorter stature. Consulting the correlations between 1MSTST repetitions and knee extension torque in our work does not imply that the individualised 1MSTST is less dependent on strength than the standardised 1MSTST, as could be hypothesised from the tendency towards lower leg fatigue. Correlations were largely similar between the two modalities. As such, we conclude that across samples and populations, the individualised 1MSTST leads to more equal strength demands.

This study has some limitations. (i) We applied convenience sampling, resulting in a young sample with a high overall 1MSTST repetition count. Very recent work investigating people with chronic obstructive lung disease implies that the effects are attenuated when the repetition count is lower (Zumbrunnen et al. 2022). Therefore, more studies applying our methodology in samples with various performance thresholds are encouraged. (ii) We used handheld dynamometry to assess muscle torque. While the gold standard to quantify muscle torque is isokinetic measurement (Chopp-Hurley et al. 2019), we standardised our dynamometry to the highest amount possible and had a single assessor performing all measurements. (iii) The repeated 1MSTSTs are a potential source of accumulating fatigue and, hence, impaired performance. However, we applied a randomised crossover protocol and controlled for a possible sequence effect in the statistical analysis. Finally, we aimed to keep the protocol as translatable as possible and did not standardise foot position and angle, as this would exacerbate preparation time. Nevertheless, foot position might influence performance (Janssen et al. 2002; Kawagoe et al. 2000; Stevens et al. 1989).

## Conclusion

In conclusion, we found that an individualisation of the 1MSTST start position to 90° knee flexion angle leads to more repetitions compared to the common start position. The higher repetition count is explained by controlling for differences in tibia length. We recommend individualisation of the 1MSTST, enabling more valid comparisons across populations and study samples. Studies quantifying the differences comparing the standardised 1MSTST to the individualised 1MSTST in various clinical populations are encouraged.

## Data Availability

The datasets generated during and/or analysed during the current study are available from the corresponding author on reasonable request.

## Abbreviations

1MSTST: 1-minute sit-to-stand test
bpm: Beats per minute
CI: Confidence interval
Nm: Newton metres
SD: Standard deviation
SpO_2_: Peripheral oxygen saturation
STS: Sit-to-stand
